# Characterization of a virulence factor in *Plasmodiophora brassicae*, with molecular markers for identification

**DOI:** 10.1371/journal.pone.0289842

**Published:** 2023-09-14

**Authors:** Afsaneh Sedaghatkish, Bruce D. Gossen, Mary Ruth McDonald

**Affiliations:** 1 Department of Plant Agriculture, University of Guelph, Guelph, ON, Canada; 2 Agriculture and Agri-Food Canada, Saskatoon Research and Development Centre, Saskatoon, SK, Canada; New South Wales Department of Primary Industries, AUSTRALIA

## Abstract

Symptom severity on differential host lines is currently used to characterize and identify pathotypes of *Plasmodiophora brassicae*, which is an obligate, soil-borne chromist pathogen that causes clubroot disease on canola (*Brassica napus*) and other brassica crops. This process is slow, variable and resource intensive; development of molecular markers could make identification of important pathotypes faster and more consistent for deployment of cultivars with pathotype-specific resistance. In the current study, a variant of gene *9171* was identified in the whole-genome sequences of only the highly virulent pathotypes of *P*. *brassicae* from around the world, including the new cohort of virulent pathotypes in Canada; its presence was confirmed using three KASP marker pairs. The gene was not present in the initial cohort of pathotypes identified in Canada. The putative structure, domains, and gene ontogeny of the protein product of gene *9171* were assessed using on-line software resources. Structural analysis of the putative protein produced by gene *9171* indicated that it was localized in the cytosol, and likely involved in cellular processes and catalytic activity. Identification of gene *9171* represents a potentially useful step toward molecular identification of the pathotypes of *P*. *brassicae*.

## Introduction

*Plasmodiophora brassicae* Woronin is an obligate, soil-borne pathogen that causes clubroot on canola (*Brassica napus* L.) and other brassica crops [1, 2]. Growing resistant cultivars is an effective approach for clubroot management. However, virulent pathotypes of *P*. *brassicae* that can overcome genetic resistance often emerge rapidly, as has recently been observed in clubroot-resistant canola in Canada [3]. The first generation of clubroot-resistant canola cultivars in Canada was effective against the initial cohort of predominant pathotypes in Canada, but resistance broke down rapidly [3, 4]. A second-generation of clubroot-resistant cultivars based on several different genes for resistance is being developed, but each new cultivar will almost certainly not be resistant to all of the pathotypes that are now present in the region. Rapid and accurate identification of the predominant pathotype present in each field is needed to help growers select the cultivar with specific resistance that will be effective against the specific pathotype(s) present in that field.

At present, identification of pathotypes is based on their virulence on selected differential host lines [4–7]. This approach is time-consuming, resource-intensive, and the results can be inconsistent. Molecular markers could make identification of pathotypes much faster and more consistent. A recent study showed that dot-blot hybridization and PCR could be used to differentiate pathotype 5 (characterized using Williams’ differential system) from pathotypes 3 and 8 using primers specific for gene *Cr811*, which might serve as a molecular marker for pathotype 5 [8]. Also, molecular markers have been used to differentiate among pathotypes 7, 11, 9, and 4 in China [9] and pathotype 5X from canola in Canada [6]. Identification of molecular markers that are pathotype- specific and independent of the geographic location is needed to facilitate the optimum deployment of resistance genes in brassica crops.

The objective of the current study was to identify SNPs associated with pathotypes of *P*. *brassicae* that were virulent on the first generation of clubroot-resistant canola cultivars in Canada based on the genome sequences of all *P*. *brassicae* samples available through GenBank and to develop molecular markers to differentiate these highly virulent pathotypes from the initial cohort of pathotypes present in the region.

## Results

### Assessment of existing molecular markers

A previous study identified differences in the partial alignments of the 18S and ITS regions between six samples of pathotype 5X (a new, highly virulent pathotype) from Alberta, Canada versus 12 samples of the initial cohort of pathotypes from Canada [6]. The differences between these two groups were used to design molecular markers to identify pathotype 5X [6]. In the current study, regions targeted with molecular markers in the previous study (shown in yellow brackets) were compared to the sequences of a collection of 5X (designated as LG3-5X) from Alberta (S1 Fig) and a highly virulent, 5X-like collection from Quebec designated as Normandin-QC-5-like (S2 Fig) [6]. The sequence of LG3-5X from Alberta was identical to 5X from the previous study (S1 Fig), but the sequence of Normandin–QC-5-like from Quebec was similar to the sequence of the initial pathotypes (S2 Fig). This indicated that the existing molecular marker might work well for collections from Alberta, but would not detect the highly virulent pathotype 5-like in collections from Quebec. Also, the molecular markers developed to detect pathotypes 7 [9], 11, 9, and 4 in China [10] did not group our 43 *P*. *brassicae* collections [11] based on pathotype (S3 and S4 Figs).

### Identification of virulence-specific SNPs and validation using a KASP assay

**Fig 1:**
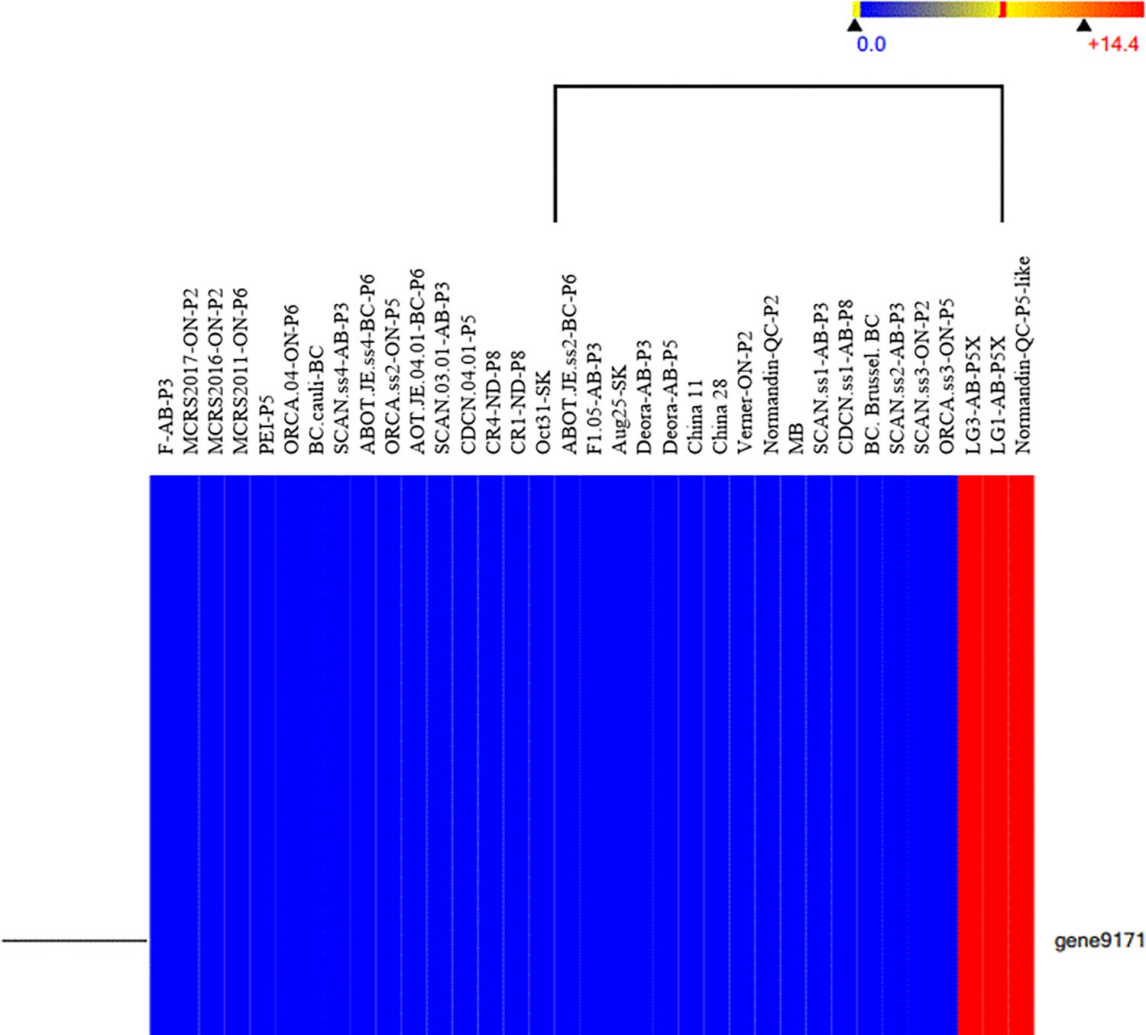
Heat map of distribution of SNPs in gene *9171* present in 35 whole-genome sequences of *Plasmodiophora brassicae*. Red color indicates the presence of important SNPs that were present only in the new virulent pathotypes; blue indicates the absence of these SNPs, which were not detected in the initial pathotypes identified in Canada.

**Fig 2:**
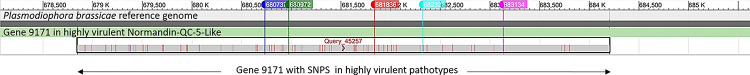
Diagram of the gene *9171*. SNPs in highly virulent Normandin-QC-5-like are marked in red. The 5 SNPs targeted for the KASP assay were marked with different colors showing the location of the SNPs in the gene.

Effector candidates and candidate genes associated with pathotype specificity were compared among 43 *P*. *brassicae* collections. The pathotypes were from across Canada from the east coast (PEI -5) to the west coast (BC-Brussel-BC-P6) and also included two collections from North Dakota, U.S.A. (CR1-ND-P8 and CR4-ND-P8) and five collections from China. None of these candidate genes grouped the collection based on the pathotype (data not presented). To search for a gene associated with virulence, SNPs were assessed in the whole genome of the 36 *P*. *brassicae* collections with known pathotypes including collections from Canada and USA, and two collections from China. Among 9,727 genes assessed, only gene *9171* (length: 5359 nucleotide) (S1 Text) carried highly disruptive SNPs that were present in all three new virulent pathotypes (LG3-AB-5X, LG1-AB-5X, and Normandin-QC-5-like) but not found in initial cohort of pathotypes when 36 collections of *P*. *brassicae* were compared (Figs 1 and 2).

In total, 70 SNPs were identified in gene *9171*, with 42 SNPs common in all three collections of pathotype 5X. BLAST analysis of these 42 SNPs against all other sequences of *P*. *brassicae* in GenBank showed that 20 SNPs were also present in other highly virulent pathotypes, including L-G2-5X, CDCN4-3X, DG3-P5X [12], and DAT 5A (S1 Table). From the 20 SNPs present in all highly virulent pathotypes, five SNPs were used to design the markers for a KASP assay (Kompetitive Allele-Specific PCR) (Table 1).

**Table 1:** Sequences and single nucleotide polymorphism used in KASP marker design and assessment.

Assay name	FAM	HEX	Sequence	Primer_AlleleX	Primer_AlleleY	Primer Common	Cycling conditions
marker-1	C	T	CGCTCCAATTCGAAAGAGTTCTCATTTGCCTCCAGCTCGAGACCCTCTATGGTCGATTTAAGCTGGGCAA[C/T]CTCTTGGGCAGCAGCATTGCCCTAAACCACTTGACTAATSAGGTACAATTTCTAGTAAA	CTATGGTCGATTTAAGCTGGGCAAC	TCTATGGTCGATTTAAGCTGGGCAAT	CCTSATTAGTCAAGTGGTTTAGGGCAA	61–65
marker-2	TC	CT	TTTCGGAGAACAAACGACTGAGTTCCTCTTGAAGACGATGTACTTCCTCTTGGCGATCTAGATT[TC/CT]CGAGTTCAGCTCTTACAGAATCGAGCTCCCGCTGCAGCTCRACGTTGCAAGCGCGCAGCTCCTCGA	TGTACTTCCTCTTGGCGATCTAGATTT	TACTTCCTCTTGGCGATCTAGATTC	GCAGCGGGAGCTCGATTCTGTA	61–65
marker-3	G	A	CCTTCAAGCGCTGGACTTGTTCGCGCAGGCTATTCAATTCCAACGTGGACTTGGAGCGCTCATGTTCCAACTC[G/A]AGGACTTGCTCCTGCAATTTGCATACAGTTCAGCACCAGCAAYGCACGTATGAAGCGGCGGCGAGAGGACCTCAAGCGTTTCAATT	TGCAAATTGCAGGAGCAAGTCCTC	TATGCAAATTGCAGGAGCAAGTCCTT	CGTGGACTTGGAGCGCTCATGTT	61–65
marker-4	A	C	ATCTGAGCGTTTAGTTGGGTCACAGTGGACGCCGATTCTTGTTTCAGAGCATCCTGCATTTTAAATCAGCGACATATCCTTCCAACCCTGCCCCA[A/C]ATGTACCAGTTGGCTCTCCATGGTCTCGCGM	TATCCTTCCAACCCTGCCCCAA	TCCTTCCAACCCTGCCCCAC	KCGCGAGACCATGGAGAGCCAA	61–65
marker-5	A	G	ATCACTTGCTCTCTGCCGAGCTTCATGAAGTTCCAGTGTGAGTTGTTCCAGCTCTTGTGTTGCTCCTTCAGATTCGGATGACGAATCGCC[A/G]TACTGTGTCTGACATTTTATGATCAGATATTTCATGAAGAACGATATAAGCATAATCAACTAACCTCTGCATCGGCGCGTGATGACGAG	TATCTGATCATAAAATGTCAGACACAGTAT	TCTGATCATAAAATGTCAGACACAGTAC	CAGCTCTTGTGTTGCTCCTTCAGAT	61–65

Targeted SNPs are shown inside brackets.

A Canadian differential set of hosts was developed to identify the range of highly virulent pathotypes that have occurred in Canada in the past years [13]. The pathotype number was based on the Williams’ differential set, followed by a letter to further differentiate virulence. Pathotypes 2B, 3A, 5X and 5-like were characterized as highly virulent and able to overcome the initial resistance genes in clubroot-resistant canola. Pathotypes characterized only using the Williams differential set but that could overcome this resistance were assigned the letter X [11, 14] A KASP assay (competitive allele-specific PCR) was developed that enables bi-allelic identification of SNPs at specific loci. KASP genotyping technology uses a fluorescence-based reporting system for the identification and measurement of genetic variation occurring at the nucleotide level. KASP-SNP markers ID 1, 3, and 4 differentiated several new highly virulent pathotypes (2B, 3A, 5X, and 5-like) from the initial cohort of pathotypes (2, 3, 5, 6 and 8) in Canada. These markers were validated by two groups using different equipment. These three molecular markers can be used to identify new virulent pathotypes of *P*. *brassicae* (Fig 2). The results were identical for markers 1, 3, and 4. The collections were clustered into two groups including new \ highly virulent pathotypes able to overcome the first generation resistance and initial pathotypes, which are not able to overcome the resistance. The targeted SNPs were not present in the host and soil samples, which demonstrated that these molecular markers were specific to pathotypes of *P*. *brassicae*. The results indicate that gene *9171* is likely a factor in the virulence of *P*. *brassicae*, especially the ability to overcome the original genes for clubroot resistance in canola.

### Protein structure and function prediction for gene *9171*

**Fig 3:**
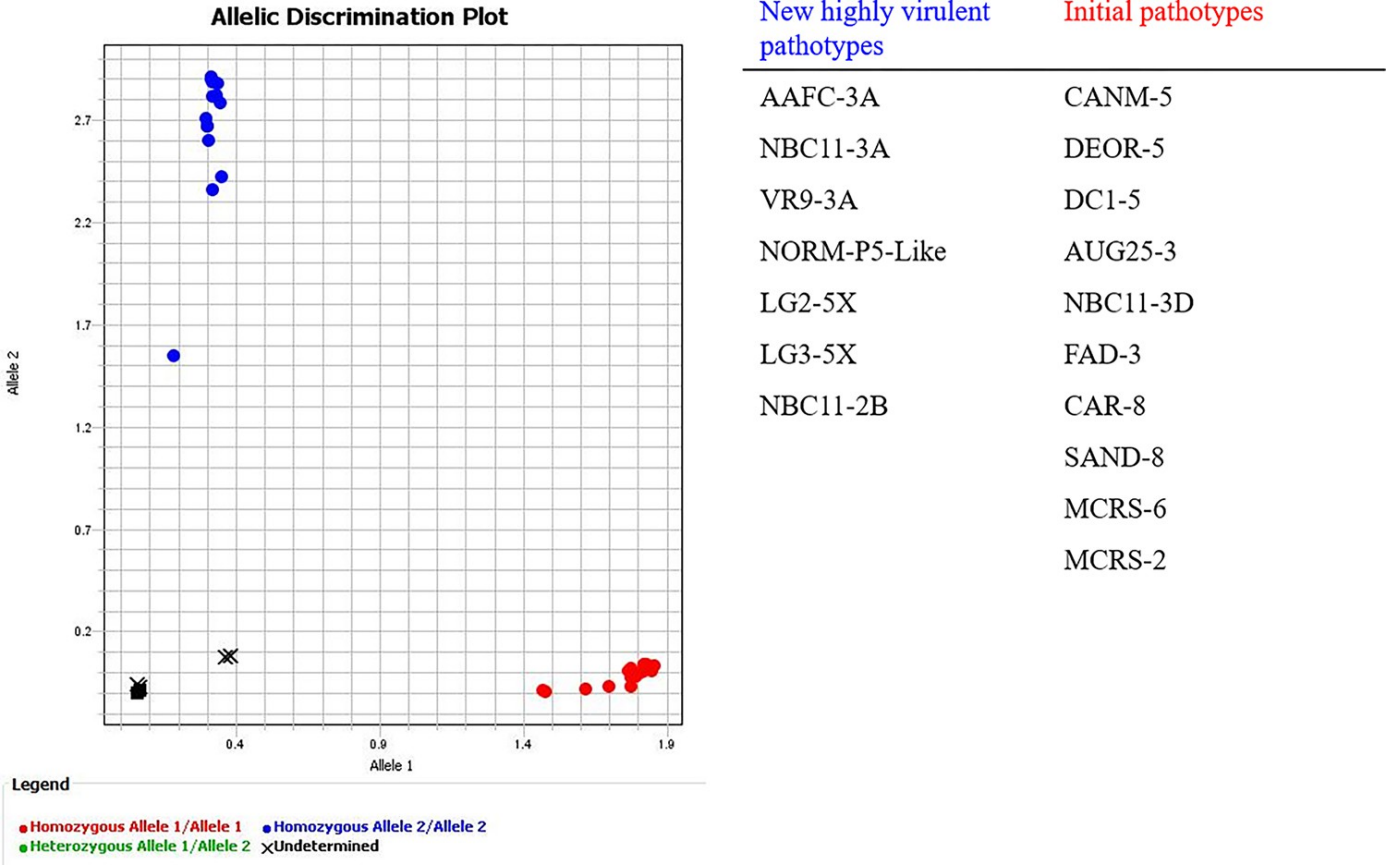
Allelic discrimination plots obtained for three molecular markers (ID 1, 3 and 4) in KASP genotyping assays. Red dots represent homozygous genotype 1 of the new virulent pathotypes; blue dots represent homozygous genotype 2 of the initial pathotypes; black squares represent the no-template control and black crosses indicate plant and soil samples.

**Fig 4:**
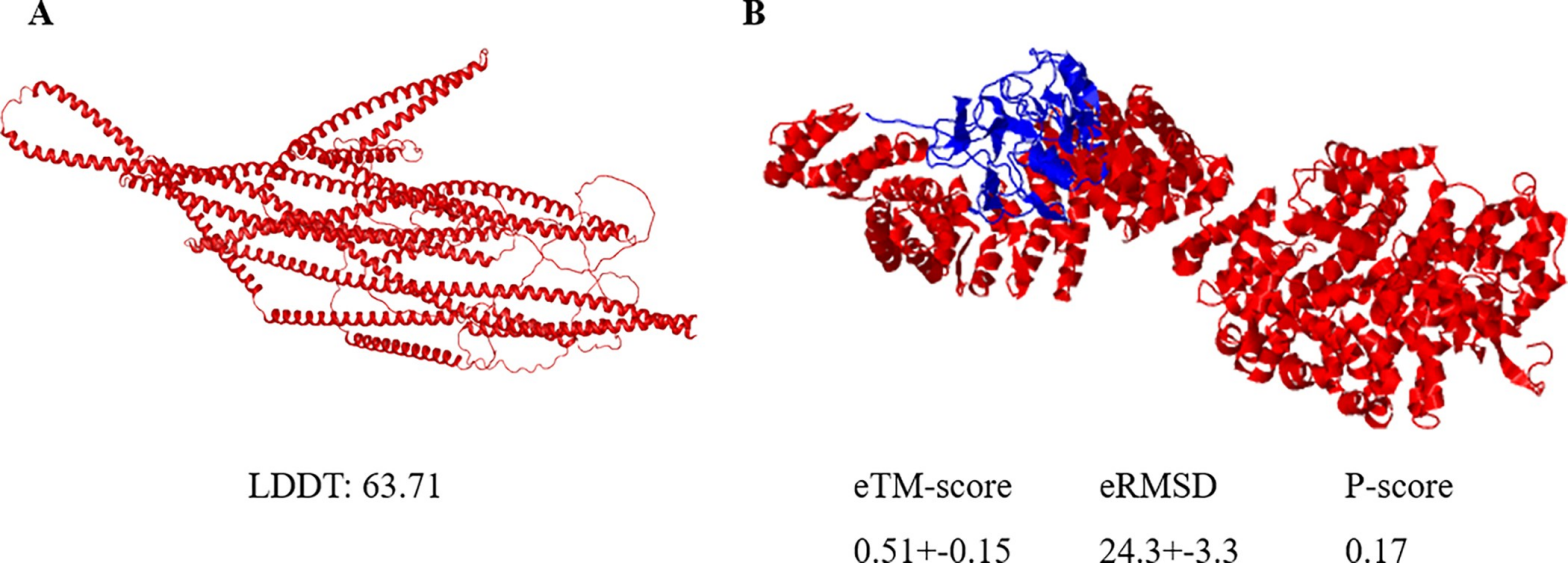
Three-dimensional structural models of PBRA-003102 protein. (A) the best structural model generated using NovaFold AI, and (B) the best multi-domain prediction using I-TASSER-MTD, where domain 1 is illustrated in red and domain 2 in blue (LDDT of 100 is best, eTM-score and P-score range from 0 to1).

The sequence of gene *9171* and the sequence of protein PBRA-003102 coded by this gene [1] did not match any gene or proteins with a known function in BLAST searches of GenBank, so the structure and function of gene *9171* could not be estimated using this approach. However, prediction of a protein’s structure can indicate its function, localization and protein-protein interaction. The RNA sequence of the PBRA-003102 protein [1] (https://www.ncbi.nlm.nih.gov/protein/857970426) was used to predict its structure and function with NovaFold AI (Fig 3A) and I-TASSER-MTD (Fig 4B) software. Three models were generated using NovaFold Al and the best model (Predicted Local Distance Difference test, LDDT = 63.7) was chosen for additional analysis (Fig 4A). Five models were obtained from I-TASSER-MTD and the best model was identified based on Template Modeling ™ and *P*-scores (Fig 4B), with an eTM score of 0.51, an eRMSD(Å) (estimated Root-Mean-Square Deviation of atomic positions) of 24.3, and a *P*-value of 0.17, where a TM score > 0.5 indicates a model of correct topology and TM < 0.17 indicates random similarity.

COFACTOR software was used to estimate the molecular function, cellular component and biological processes of the PBRA-003102 protein. The top 10 closest structures in the Protein Database Bank (PDB) were identified using TM-align software (S5 Fig). The best structure match was a 7WKK structural protein from the nuclear pore complex of *Xenopus laevis* (PDB DOI: 10.2210/pdb7WKK/pdb) (S5 Fig).

Gene Ontology (GO) terms were predicted for cellular components (S6A Fig), biological processes (S6B Fig), and molecular function (S6C Fig). GO analysis indicated that the PBRA-003102 protein was involved in cellular processes (Cscore 0.86) and might have catalytic activities (Cscore 0.43). However, the function of this protein could not be accurately predicted solely based on sequence alone methods.

## Discussion

Several groups have previously attempted to identify candidate genetic markers [9, 10, 12, 15] and effectors [1, 15–17] that could be used to identify pathotypes of *P*. *brassicae*. For example, five molecular markers were reported to differentiate several pathotypes from China [9] and SNP sequences have been reported for molecular identification of pathotype 5X from Alberta [6] However, the obligate nature of the pathogen and the paucity of genome sequences from multiple pathotypes has made progress in identification of specific alleles and marker development very difficult [18, 19]. Previous attempts to identify markers from the limited published data available have been unsuccessful [18, 19]. As in these previous studies, published markers and effectors genes were not associated with specific pathotypes in the current study. Also, the genetic sequences identified by the five Chinese markers did not cluster the GenBank collections based on pathotype in the current study. This might be due to the absence of these pathotypes in the *P*. *brassicae* collections available in GenBank. Similarly, the SNP sequences used to identify pathotype 5X from Alberta [6] were not found in the genome of a pathotype 5-like collection from Quebec (S2 Fig).

In a previous whole-genome study of *P*. *brassicae* collections from different locations, similarity analysis grouped collections based on geographic origin rather than pathotype [11]. It is possible that differences have accumulated within the specific virulence factors associated with individual pathotypes from different regions worldwide. If so, molecular markers selected to identify specific pathotypes might only be effective regionally and could not be used to identify similar pathotypes from other regions. Therefore, it is important to confirm the presence of target SNPs for molecular identification in samples from a range of locations and countries when developing markers for a specific pathotype. In the current study, gene *9171* and associated SNPs were identified as being associated with highly-virulent pathotypes on canola (S1 Table). KASP analysis demonstrated that the SNPs in gene *9171* were associated with a cohort of new, virulent pathotypes in Canada and so may be associated with the virulence of *P*. *brassicae* that is able to overcome the first clubroot resistance genes in canola.

The function of gene *9171* could not be determined with certainty. A comparison of its amino acid sequences with the database did not identify any similar proteins and prediction based on structural analysis did not assign a clear biochemical or biological role to this gene. The large size of the gene and the presence of two subunits make prediction analysis challenging. However, analysis indicated that gene *9171* is localized in the cytosol and involved in cellular processes and possible catalytic activity. No signal peptide or transmembrane was predicted for this protein. Studies of the pattern and timing of expression of gene *9171* are needed, but the obligate nature of this pathogen greatly increases the difficulty of such studies.

Assessing more markers located in the putative region of gene *9171* using KASP might identify additional markers for identification of pathotypes. The SNPs used in this assay were not present in collections of initial pathotypes from the USA, China, the published reference genome from Europe [1], or the initial cohort of pathotypes from Canada. However, there are currently no sequences of highly virulent pathotypes from outside Canada available to confirm the presence of these SNPs in other countries to validate the KASP assays for other regions. However, the three KASP markers identified could be used to bypass and thereby improve portions of the current deferential set method by making it less expensive, less time consuming, and more consistent.

## Materials and methods

### Identification of SNPs associated with virulence

SNPs associated with virulence were identified in the whole-genome variants of 36 collections of *P*. *brassicae* obtained from a previous study [11]. Heat maps based on genome-wide SNPs were constructed using Euclidean hierarchical distance with centroid linkage as the clustering algorithm in the ArrayStar component of the Lasergene 15 software package (DNASTAR, Madison, WI). Gene *9171* (CDSF01000144.1:678875–684233 *Plasmodiophora brassicae* genome assembly pbe3.h15, scaffold scaffold_8) was visualized using GenVision / Genome Mapping software (DNASTAR). Also, the consensus sequences of gene *9171* in the highly virulent pathotypes CDCN4-3X, DG3-P5X, LG1-AB-5X, LG2-5X, LG3-AB-5X (all from Alberta, Canada) and Normandin-QC-5-Like (a highly virulent collection of pathotype 5 from Quebec characterized using the Williams’ system) were analyzed by BLAST comparison with the NCBI nucleotide database (www.ncbi.nlm.nih.gov/BLAST/) and the regions of similarity among these collections were identified. Among the 20 SNPs that were present in all highly-virulent pathotypes, five SNPs were chosen based on the surrounding SNPs and the required length for KASP assay design.

Nineteen of the *P*. *brassicae* collections (Fig 3) were available for additional assessment. For each collection, a single clubbed root was surface-disinfected with 30% household bleach for 10 min followed by 90% ethanol for 1 min and washed 3 times with deionized water. Suspensions of resting spores from the club were prepared by homogenizing each club separately in a commercial blender and filtrating the blended material through cheesecloth to remove large plant fragments [20]. Each spore suspension was then passed through a filter with 50 μm pore size and then centrifuged at 1000 g for 10 min to remove plant debris and soil.

A DNeasy® PowerSoil® Kit (Qiagen, Maryland, USA) was used for the extraction of *P*. *brassicae* DNA following the manufacturer’s instructions. The DNA of non-inoculated Shanghai pak choi roots (*B*. *rapa* var. *chinensis*) cv. Mei Qing Choi (Stokes Seeds, St. Catharines, ON, Canada) and non-inoculated soil was also extracted as a control.

### KASP assays

For each putative SNP, KASP™ assays using two allele-specific forward primers and one common reverse primer were designed (LGC Biosearch Technologies, UK) based on the annotated SNP sequences. Molecular markers were genotyped and validated for 19 *P*. *brassicae* samples plus plant and soil controls by LGC genomics. The KASP assay and molecular markers were validated for the second time using KASP-TF Master Mix (LGC Biosearch Technologies, UK) and Applied Biosystems QuantStudio 6 Flex Real-Time PCR System in our laboratory. The KASP thermal cycling conditions included the pre-read stage (30°C for 1 min), hold stage (94°C for 15 min), touchdown stage (94°C for 20 s and 61°C for 60 s (drop by—0.6°C/per cycle) for a total of 10 cycles achieving a final annealing temperature of 55°C). Followed by the PCR stage (94°C for 20 s and 55°C for 60 s, repeated for 25 times (a total of 26 cycles). The KASP post-read stage was 30°C for 1 min. The plate can be recycled to perform a second post read at 94°C for 20 s and 57°C for 60 s for a total of three cycles. Genotyping data were viewed on Allelic Discrimination Plot.

### Protein structure and function prediction

The RNA sequence of the PBRA-003102 protein [1] coded by gene *9171* was obtained from GenBank. NovaFold AI software (DNASTAR) was used to predict, visualize and analyze the protein structure using the AlphaFold 2 method. To confirm the structure, the multi-domain protein structure and function prediction of this protein was obtained using C-I-TASSER On-line Server and I-TASSER-MTD on-line server (available online through the Zhang lab from University of Michigan) [21]. The COFACTOR algorithm (I-TASSER_FUNCTION) was selected for this analysis because it had previously been ranked as the best method for protein function prediction in community-wide CASP9 experiments [21–23]. The biological functions of the query protein were derived from the structure model using COFACTOR software [21].

The structural model of each individual domain was constructed independently by I-TASSER (available online through the Zhang lab from University of Michigan). The individual domain models were assembled into full-length structures using DEMO software [21] (https://zhanglab.ccmb.med.umich.edu/DEMO/) under the guidance of quaternary structural templates and deep-learning distance profiles. The protein functions at both the domain and full-chain levels were annotated using COFACTOR based on structures, sequences, and protein-protein interaction networks. The accuracy of three models generated by NovaFold Al was quantitatively evaluated using the LDDT test. LDDT ranges between 0 to 100, where 100 = best; the model with the highest LDDT score was selected for further analysis. The accuracy of predicted domains was estimated using TM-score (eTM-score) plus eRMSD. These estimates were calculated based on the significance of the structural analogous templates for domain model assembly, convergence parameters of the domain assembly simulations, satisfaction degrees of the inter-domain distances/interfaces and the estimated accuracy of the individual domain model. The eTM value is typically in the range of [0,1], where a higher value indicates a model with a higher degree of confidence. P-values estimate the structural similarity of the population formed in the modeling simulations based on SPICKER clustering. P-values range from 0 to 1; a higher value means the structure occurs more often in the simulation. The inter-domain interaction is defined as ≥ 1 residue pairs with a distance < 8Å apart from the linker region. The probability ranges from 0 to 1, and a large value indicates that the two domains have a high probability of interaction.

From this 3D structural model, COFACTOR searched for local and global structure matches in the BioLiP protein function database to identify functional sites and homologies. Functional insights, including gene ontology (GO), were derived from the best functional homology templates. For GO, additional insights were obtained from UniProt-GOA software based on sequence and sequence-profile alignments and from STRING software based on protein-protein interaction inference (available online through the Zhang lab from the University of Michigan).

Protein subcellular localization and signal prediction was performed using different online servers including BaCeLLO [24], DeepLoc-1 [25], DeepMito [26], DeepSin [27], MULocDeep [28], Phobius prediction [29], SCLpred-EMS [30], TargetP-2 [31], BUSCA [32] and TMHMM [33] (S2 Table).

## Supporting information

S1 Fig Comparison of 18S (A) and ITS (B) regions in *Plasmodiophora brassicae* reference genome GCA_001049375.1(initial pathotype), L-G3 (virulent pathotype 5) sample from Alberta sequenced by [11] and L-G3 (Virulent pathotype 5) sequenced by [6]. The yellow highlight bracket is the targeted sequence for molecular marker development.
(TIF)

S2 Fig Comparison of 18S (A) and ITS (B) regions in *Plasmodiophora brassicae* reference genome GCA_001049375.1(initial pathotype), Normandin (virulent pathotype 5) sample from Quebec sequenced by [11] and L-G3 (Virulent pathotype 5) sequenced by [6]. The yellow highlight bracket is the targeted sequence for molecular marker development.
(TIF)

S3 Fig Genes (A) 9559, (B) 303, and (C) 533 used for the development of the molecular markers for pathotype 7 from China did not group 43 collections of *P*. *brassicae* based on the pathotype. SNP disruption is represented with color codes. Red color represents non-synonymous SNPs compared to synonymous SNPs in blue.
(TIF)

S4 Fig Genes 444, 9348, 8439, and 2543 used for the development of the molecular markers for pathotypes P4, P9, and P11 from China [10]. SNP disruption is represented with color codes. Red color represents non-synonymous SNPs compared to synonymous SNPs in blue.
(TIF)

S5 Fig Top 10 structural analogs in PDB as identified by TM-align. Query structure is shown in cartoon, while the structural analog is displayed using backbone trace. Ranking of proteins is based on TM-score of the structural alignment between the query structure and known structures in the PDB library. RMSD^a^ is the RMSD between residues that are structurally aligned by TM-align. IDEN^a^ is the percentage sequence identity in the structurally aligned region. IDEN^a^ is the percentage sequence identity in the structurally aligned region. Cov. represents the coverage of the alignment by TM-align and is equal to the number of structurally aligned residues divided by length of the query protein.
(TIF)

S6 Fig Gene ontology terms and Cscore^GO^ values for: A) cellular component, B) biological process and C) molecular function. Cscore^GO^ values are the confidence score of the predicted terms (range 0–1, higher value indicates more confidence). The Cscore^GO^ of each component is color-coded using the scale on the bottom-left.
(TIF)

S1 Text DNA sequence of gene *9171* in *Plasmodiophora brassicae*. (DOCX)

S1 Table Twenty single nucleotide polymorphism in gene 9171 present in new pathotypes of *P*. *brassicae*. The SNPs and resulting changes as well as the SNP location on the gene *9171* is presented.
(DOCX)

S2 Table Software programs used to determine the location of gene *1971* of *Plasmodiophora brassicae*. (DOCX)
