# Supplementary figures and images for: Characterization of a virulence factor in Plasmodiophora brassicae, with molecular markers for identification
Source: PLoS One. 2023 Sep 14;18(9):e0289842. doi: 10.1371/journal.pone.0289842 (PMC10501564; doi:10.1371/journal.pone.0289842)

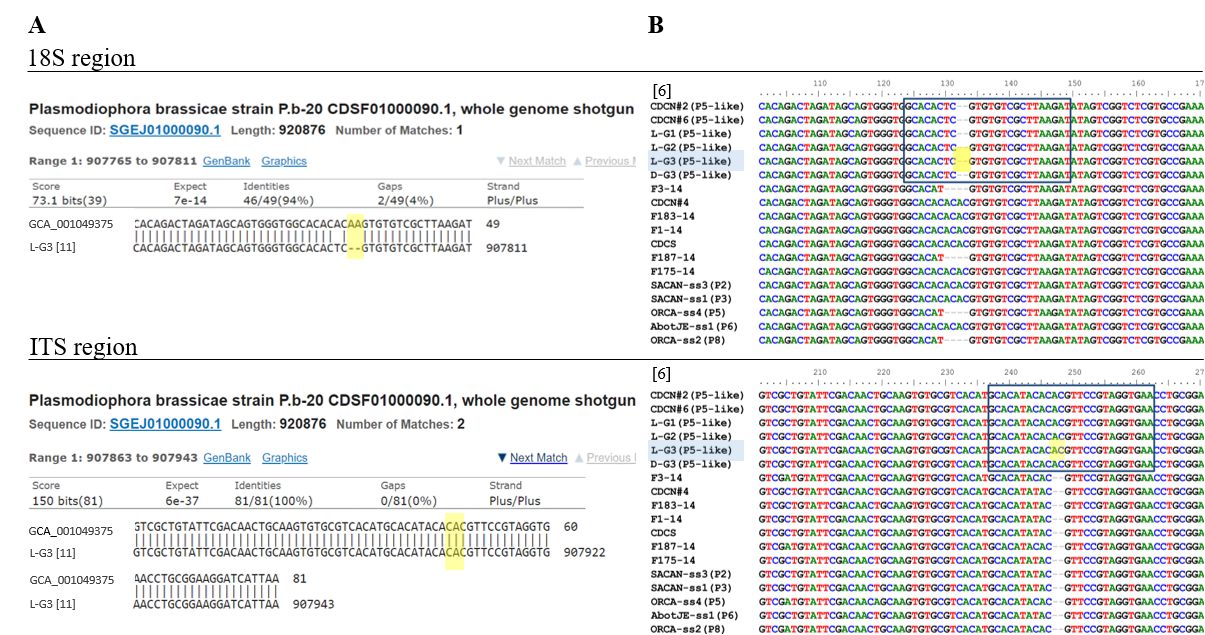

Supplement: S1 Fig — Comparison of 18S (A) and ITS (B) regions in Plasmodiophora brassicae reference genome GCA_001049375.1(initial pathotype), L-G3 (virulent pathotype 5) sample from Alberta sequenced by [11] and L-G3 (Virulent pathotype 5) sequenced by [6]. The yellow highlight bracket is the targeted sequence for molecular marker development. (TIF) [file pone.0289842.s001.tif]

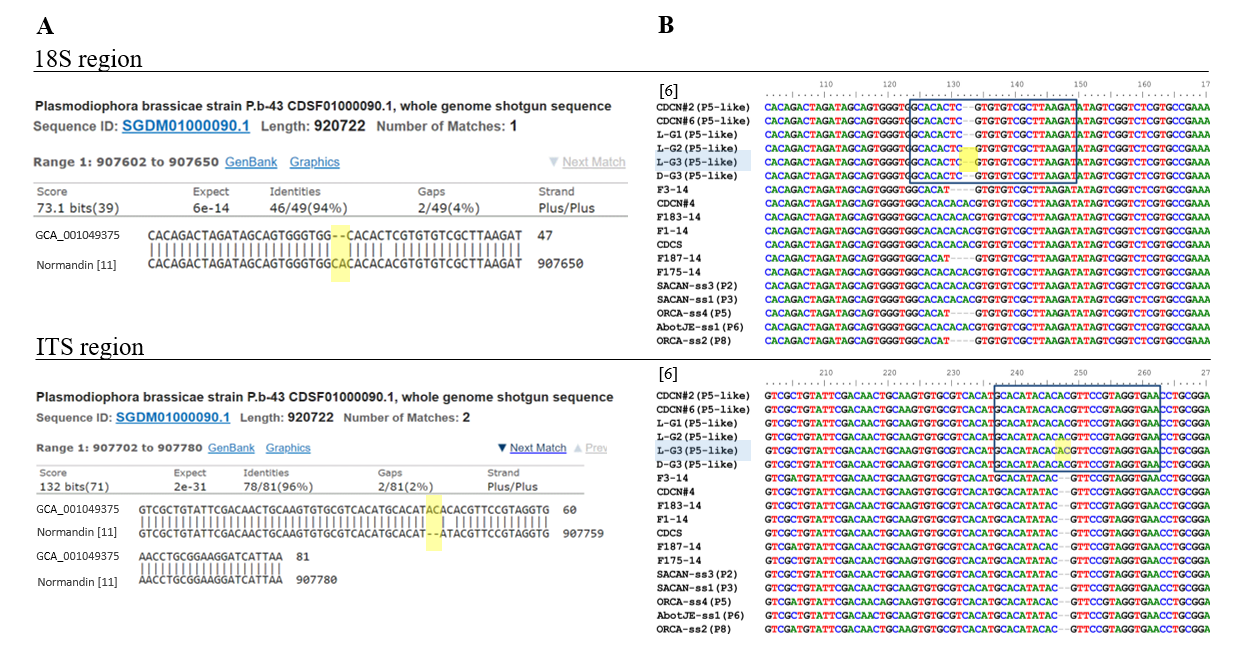

Supplement: S2 Fig — Comparison of 18S (A) and ITS (B) regions in Plasmodiophora brassicae reference genome GCA_001049375.1(initial pathotype), Normandin (virulent pathotype 5) sample from Quebec sequenced by [11] and L-G3 (Virulent pathotype 5) sequenced by [6]. The yellow highlight bracket is the targeted sequence for molecular marker development. (TIF) [file pone.0289842.s002.tif]

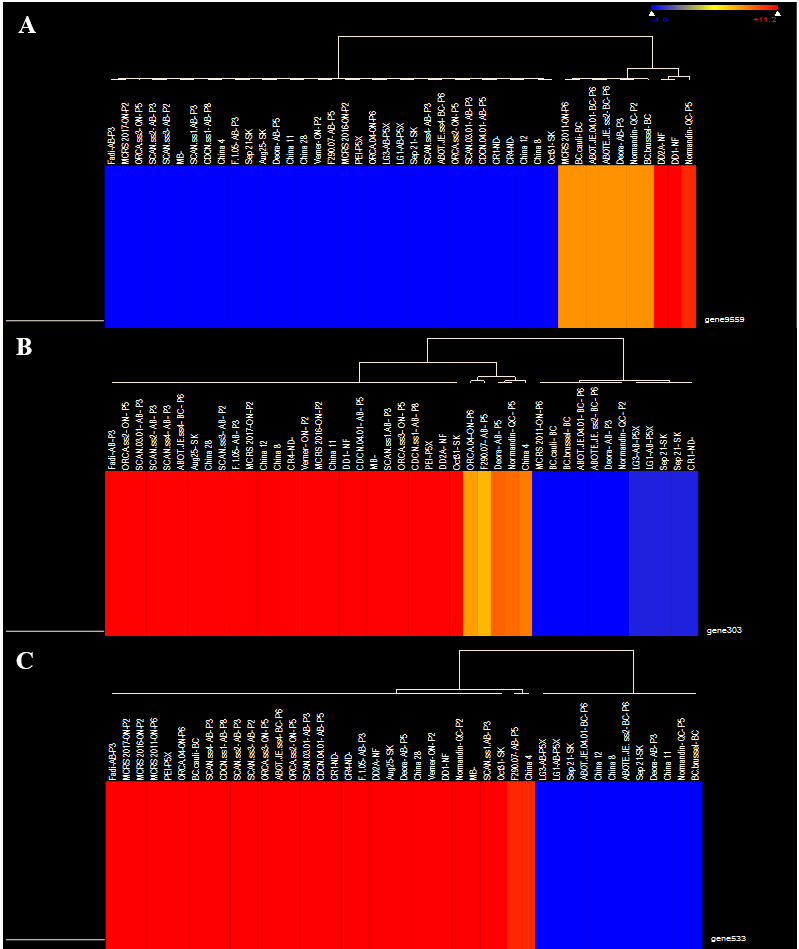

Supplement: S3 Fig — Genes (A) 9559, (B) 303, and (C) 533 used for the development of the molecular markers for pathotype 7 from China did not group 43 collections of P. brassicae based on the pathotype. SNP disruption is represented with color codes. Red color represents non-synonymous SNPs compared to synonymous SNPs in blue. (TIF) [file pone.0289842.s003.tif]

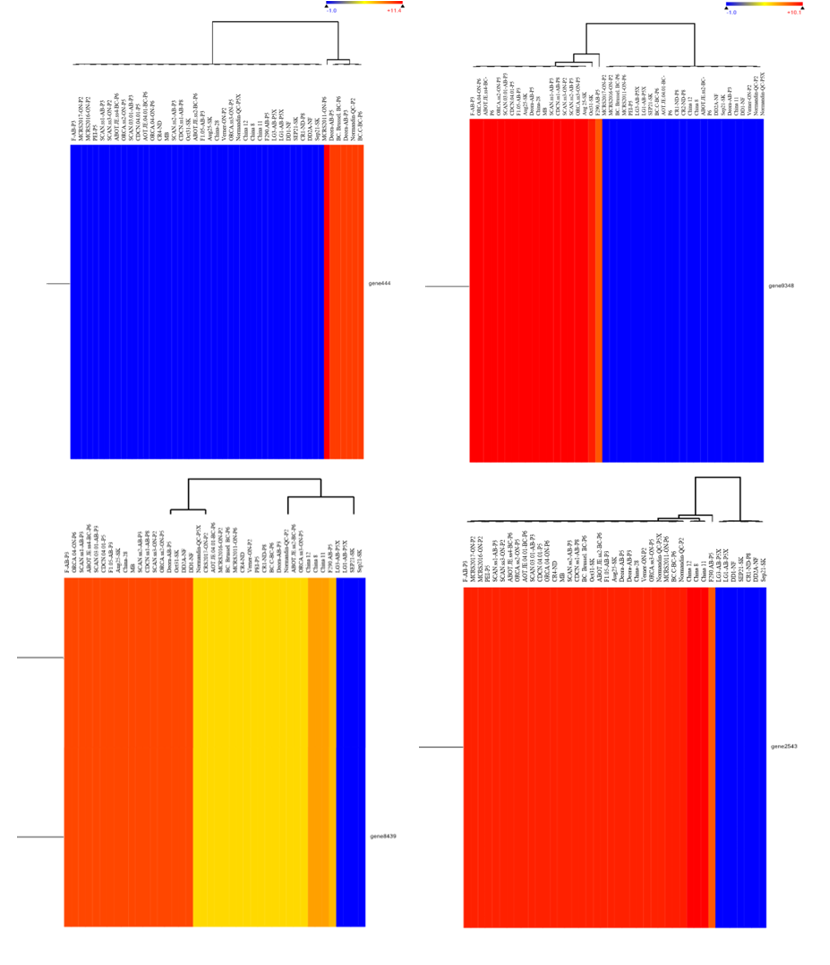

Supplement: S4 Fig — SNP disruption is represented with color codes. Red color represents non-synonymous SNPs compared to synonymous SNPs in blue. (TIF) [file pone.0289842.s004.tif]

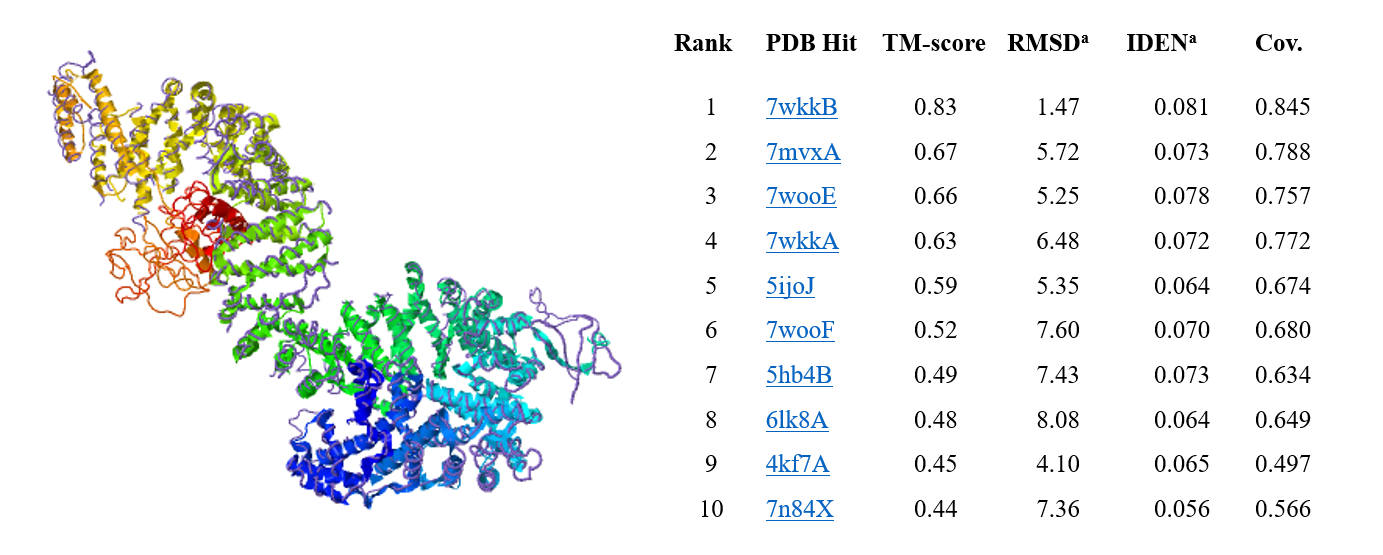

Supplement: S5 Fig — Query structure is shown in cartoon, while the structural analog is displayed using backbone trace. Ranking of proteins is based on TM-score of the structural alignment between the query structure and known structures in the PDB library. RMSDa is the RMSD between residues that are structurally aligned by TM-align. IDENa is the percentage sequence identity in the structurally aligned region. IDENa is the percentage sequence identity in the structurally aligned region. Cov. represents the coverage of the alignment by TM-align and is equal to the number of structurally aligned residues divided by length of the query protein. (TIF) [file pone.0289842.s005.tif]

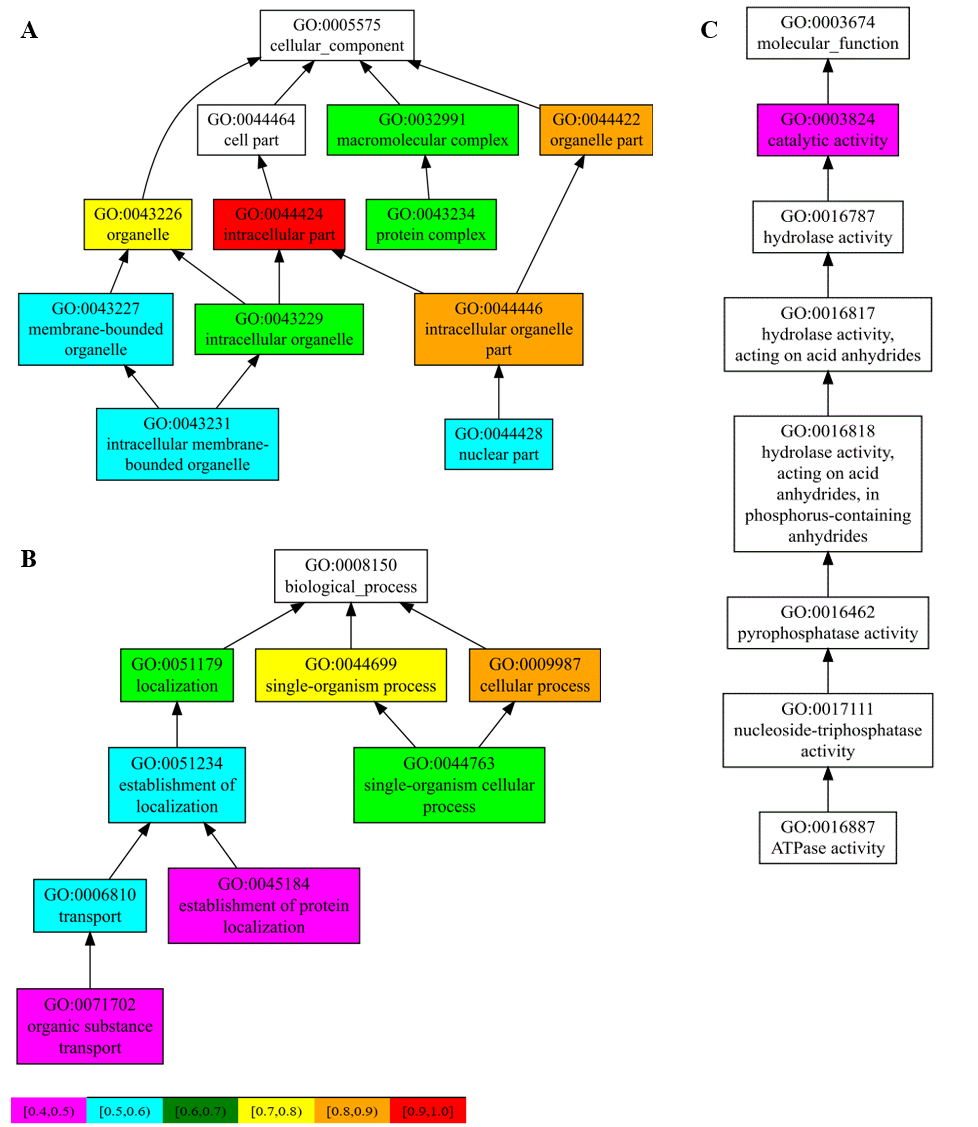

Supplement: S6 Fig — Gene ontology terms and CscoreGO values for: A) cellular component, B) biological process and C) molecular function. CscoreGO values are the confidence score of the predicted terms (range 0–1, higher value indicates more confidence). The CscoreGO of each component is color-coded using the scale on the bottom-left. (TIF) [file pone.0289842.s006.tif]
