# Supplementary material for: Characterization of a virulence factor in Plasmodiophora brassicae, with molecular markers for identification
Source: PLoS One. 2023 Sep 14;18(9):e0289842. doi: 10.1371/journal.pone.0289842 (PMC10501564; doi:10.1371/journal.pone.0289842)
Supplement: S1 Text — (DOCX) [file pone.0289842.s007.docx]

GenBank Graphics

>CDSF01000144.1:678875-684233 Plasmodiophora brassicae genome assembly pbe3.h15, scaffold scaffold_8, whole genome shotgun sequence

TCACGTCAACGATATGATAGCGGATGCAACACCGACGGCCGTCGCGATCCTGGCAAGGCGGAGCCGTTAGAAAAAGCTGGAAGCCGCTTTCGCATGGCGAGGTCTCCTTACCCGTAAACAGCTCTAGACACTGGGATAGTGATTGCCGACGGGCGGTCGTCGCAGCCGCCGGACGCAAGCTTATCGCGAACCGAGTGACTCATAACGCGAACTTTGTCTAGGATCTGGCGTTTCTCGTACCGGAGCTCAGCGACCATAGAATGCAGCATCACCACGACCGCTGCGTCGGATCGAGGCAGCATGTCGATGGAAGAGATCGGCAACGGGCTGTACTCTCCTGGAGGAACAGACACAGCTTTCGCTGGCAATGCTTGCACTGGGCCGATGGAAGACGAAACCGACGGTGCAGGTGCGGCAGCGCTGGGCAACGATATGCCATCCGGAAGCGCATAGCGGGAGGCCGTACCAGTACGGCGTGTGAGACCGGCACCACCTGCAGGAGTCGGACGAGGACCGGTTGCTGGAGTAGACGCAGAAGATGACGGTCGTGGAGGTGGAAGCGCAGGCTTGCTCGGCTCTGATGCTGGCGCGTTGACGTCGACATACTTTTTGAGGACTGGATCATACACGAACTGAGTGTCCTCAGTCTGCAATGACAGTAAGCATTCTCGAACGACGTTCGATTCAAACGTAAATAATACCATGTCAGCGATAGCAATTGGCTTGCCGTTCTCGTCATACAACTGCCGGCGGAACAGTTTTGTGAAGGACTGTAATAGAGACGATGACCTAGGCTTAGATGGGGCGGCCGACGTTGCTGGCGGCGACTTCGCAGCGGCCTGAATCGTTGAACGGGTGTCGGGTTCTTTGTCGCTTGAAGCTTGCCTGCCACTTTGCAGCTTCTCGAGCGCGATAATATAGTTCTCTGTACAGCAGCCCTTAGCATACGACCATGGGTTAAAACATGGTTCAAATACCTCGTAGCTCCGCGTACAACCCGACAAGCGTGATGGTTCGGCGTTCAAGGTCGCGATACTTCTCTTCAAACTCAGAAGTAGGTTTAGGTGTGTGGTCCTCACGAGCATCCGCTGCCAGACGCACTGGATTGCGCGGAATCGCAGCAGCTGGTGGCGCTCGTAACGGTACCGCCGATCCAGGAGCATGACTTCGAATGCGCTGGGTATTCTGGCTAGCCTTCAGTTCTTGTAGCTGCGTGGTGAGATCCGTGATCTGCTGCTTCAGACCAGAGATCTCAATTTCGTAGGCTTCACGAATAGCGCCACCTTGAGTCGGCGACGGCGCTTTGTGCGGCTTGTCTTCTTTAGAACGCTGACTCAGCTCCACTGCCTTCTCCAATTCTTCGCGAAGAGAAGCCAACTCCTTTTCCAATTTCTTCCTTTGCTTGGTTGCTGCGTGACGTTCATCTTTAATGGACGCTTCCTTTTCCTTCAGCAGGCCACGCAGTGTCTCGACTTCTTCAGCCCTATCCGCCAACTGCGCTTGAACGTCATCGGTTCTCAATGCAGACTTCATCATATCGAGCTGCTCAGTGACATCGTCGAGCTTGTCGCGCAGGCACTTGTTTTCCGCGGACATGGCATCAAGCAAGGCAACCTTTTCCTGCAAAGAGCCCAACTCCTTCCTTCGGCTGGCAAGTTCCTCGTTCGTCACCGATAACGAAGAGCTCAGTTCGTGAAGTTTCTTCTGTAGCGCCTTCGACGACTGTACAGCGTTGTCAAGTTGCAGTTGCTTTTGACGAAGCTCCTCGCGAAGTGCGGTTTCCCTGGCTCGTGACGCTTCTTGGGAATCTAAGGAATTCCCCTTCAAGCGCTGGACTTGTTCGCGCAGGCTATTCAATTCCAACGTGGACTTGGAGCGCTCATGTTCCAACTCAAGGACTTGCTCCTGCAATTTGCATACAGTTCAGCACCAGCAACGCACGTATGAAGCGGCGGCGAGAGAACCTCAAGCGTTTCAATTTGCAGCTGAGACTCAACATCGTTTTTCGCCTGAAGATGCTTGAGCTCTCGTTGCAAACGTGCGTTGGCGTCACGGAGGCGCTCCAATTCGACAGAGTTCTCATTTGCCTCCAGCTCGAGACCCTCTATGGTCGATTTAAGCTGGGCAATCTCTTGGGCAGCAGCATTGCCCTAAACCACTTGACTAATGAGGTACAATTTCTAGTAAAGTGGCTCACCAACGTACGTCAGGTAAAGAATCTGCCACGGACCGCAGCGCTTCGATTTGCAGTAGCAATTCATTGTTGCTTTGCCGAAGCGTCAGAAGCTCCTCAGGCACATCGTCCTGACGTACTTCGCGGATTTCGTATTCCACGTCATCCACCAACGAGGAACTTGAACTGCCACTGGCCTCAAGTGGCTGCGGCTGCATAAACCACCAATTATCGCAGGAACATTATCATCGTGTTCGTACACATAAAGTCCTACCTCGAATGACGTGACATGGGAAAATGAATCGCGCAACTTCGCATCGGGCACCTCCCCATTCGGCACAGCTTTGATGTTTCCATTGAGGTTTCCAGCTGATTCTTCTTGAGGACTGGCGGTGTGCTCTTTGAGAACGTTGATCTCCAGTAACAATTCAACACGTCGTTCCTCAGCTTCTTGTAATTGATTCTGCATATAACATCAACCTTAACTCCAACTACATGAGAGAGCGTACGTACCTCCAGCAACTCGACTCGAGTCTGAGCTTCAGTTCTTGCAGAATCCAGTTCTTGCTGTAACTCAAACAAGCGAGATCGCAACTCCTCGACCTCAGGAGAATCGCCCTTCAAAAACAATGAGGTCTTGCTCTGAGAATTCTCAAGCTCGTCGATTCGCGCCTGTAGCTGCTCAACTTTACTTCGCTCATCCTCCTATAATTCGCTATTAGGAAAAATACGACATCCGGAAAGCCCTCTGACATTTTCGGAGAACAAACGACTGAGTTCCTCTTGAAGACGATGTACTTCCTCTTGGCGATCTAGATTCTCGAGTTCAGCTCTTACAGAATCGAGCTCCCGCTGCAGCTCGACGTTGCAAGCGCGCAGCTCCTCGATTTCAGGAGCAGTATTATTCACGAGCAAAGGGCCTTCGTCCTGTGAGTTCATAAGATCGGGAACTTGTGACTGGAATTCTTCAGACTGTCTGCGCTCATATTCCTGCAATTTCCAGTTAGACAAACGATGGCATTTGGGGAGGCATCCTGACGTTTTCGGAAACCAAGCGGCTCAGTTCCTCCTTCAGCCGTTGTACTTCTTCTTGAAGATCTTGATTCTCTTTATCGAGGGCATCACCATCGGCAACAAGGGCGCTTCGAGCAGCTTCGACAACATTGTACTGTGTTCGCAGTTCGTCTATCTGAGCGTTTAGTTGGGTCACAGTGGACGCCGATTCTTGTTTCAGAGCATCCTGCATTTTAAATCAGCGACATATCCTTCCAACCCTGCCCCACATGTACCAGTTGGCTCTCCATTGTCTCGCGAGCATCGTAGCTAGCTGACAGCTGCGACTTGACCGATTCGAAGTCCTGCTCCGCTTGACGCAGCTGGTCTGCAATCTCGGAAATGGCATCCTCGTGTGCTTGTTCCAACTCTGCCGCGTTCGCACGAAGCTGCTCAATTTCCACACGAAGATCACGAGCATGCTCAACCTCCGCGGTCGCATTTCGCTGCAGACAGAAGCCGTCAGATAAATCGATGTGACCAACGCATGCAGCCGACCAGGTCGTCCCGAAGTGCAGCAAGCGCGCTTTCGCTCATTTCCAATTTCCTCGTAAGCGACTTGATCTCCTGTCGAGCTTTAAGGAGCTCAGCTTTGGTCGGGCGCTCTGAGGCCTTATCTAGTTTCGCTTGCAGCCGCTGGACTCTGGCACCCTGATTCATACATTAGCTCGACACGCAACATCAAACAGACAGCGTACCTGTTTGTCAACTTCAGCATGCGCTGCAACAAGCTGGGAAGTTAACCTGTCGATTAAAGAGGACTGCCGAGCAATTTCCATCTGAAGTTGTTGGAGTTCTTTGTCTGACGTCGAGGCTGTCCGCATCTGCCTGTGGCGAAGTATTTCCATCTCAGCGATCAGGTTTTTGATAGTAGCGTTCTTGGAGTCAAGCTGCTTGCGGACGAAATCGAAATTCGCTACTAGCTCTTCTTGCGCCTGCACTAATTCAAGGTGCTTCGAAAACACGAGATCGCGATCACTTGCTCTCTGCCGAGCTTCATGAAGTTCCAGTGTGAGTTGTTCCAGCTCTTGTGTTGCTCCTTCAGATTCGGATGACGAATCGCCGTACTGTGTCTGACATTTTATGATCAGATATTTCATGAAGAACGATATAAGCATAATCAACTAACCTCTGCATCGGCGCGTGATGACGAGACCTTCAGTTTCTCCTCAAGATCATCACGTTCGCCTTGAAGGTCGGCATACTCTGCCCGCAGAGTGTTGAGCTCGTTCAGTACAGATTGCAAAGCATCCCTGTCTTCGTCCTGTTTACGAATAGCGGCATCGAACTGAGTCTGTTGGCGTTCGAGCTCGTCGGCTGCCTTCTCCAGTTGACTTTTGAGAATGGTCGTTTCAGTTAAGTCAGGCTCTGTTGTAGTCTAAGGTGCAAACACATATTGCAATCAATACAAGCAAAGCCAGCACGTGTTGAACAAATAACTGACGTTGCGAGATTCCGCTACTGCCAATGCCTGTCGAAGATTATCGATATCGTCCAAGGCGCGGTTCAGATCATCTTGCGTTTCAGCTTCTCGCTGTCTAAGGATGTCCAAATCGTGTTTCGCCTTTTCGATGGCACGCACAAGGTCTTTCTTCTCCTCTGCAAGGTCGGCAATCTCCTCATCTGCAGCGTCTAATGCTTTGCGAAGTTGGCTTCTCTCCGACTCACAATCAGCAAGCTGACGGTTCGCATCGCTGAGCTCCGACGTCAGACGCTCGAACTCGTCGACGGATCGAGACGCCAGTTCAGACGAGGTGGACGATTTTCGCCTCTGCAAAGAACGCATTAGTACACTAACCACGCCTATGCGAGGGTTCAAACCTCTTCGCGGACGGCGGCTGTCGCACGTTCCAACTTCGCATTCAGGTCCTTTTCGACAGCTTGTAGACGTTTCCATTCTGGGACGAGTTTCATCAGCTTCCCTCGTAAAGCGGCGACCTGCGCCTCTGCGTCCTCTGCTCGCGACCGCTCCTTCTCGACGTCGGCGTCCGCGCGCTGTCGCTCGGCGTCGAGCTGCGCTTCGAGCTCCTTGATCACCTCCTTCTGGCGCTTCCATTCCGGCACCAGACGCCTAAGCTTCTCGCGATACTGTTCGGCCAGGTCGTCGGCAGACGACGTCGCACCCGTGGCCAT
