# Supplementary material for: Characterization of a virulence factor in Plasmodiophora brassicae, with molecular markers for identification
Source: PLoS One. 2023 Sep 14;18(9):e0289842. doi: 10.1371/journal.pone.0289842 (PMC10501564; doi:10.1371/journal.pone.0289842)
Supplement: S1 Table — The SNPs and resulting changes as well as the SNP location on the gene 9171 is presented. (DOCX) [file pone.0289842.s008.docx]

| Nucleotide change | Classification | SNP location |
| --- | --- | --- |
| T>G | Change-Non coding RNA | 679535 |
| A>T | Synonymous | 679556 |
| G>A | Synonymous | 680413 |
| A>G | No change | 680737 |
| C>T | Change- Non coding RNA | 680780 |
| A>G | Change- genic- splice | 680805 |
| C>T | Synonymous | 680824 |
| G>A | Synonymous | 680900 |
| C>A | Non synonymous | 680914 |
| T>C | Non Synonymous | 680972 |
| T>A | No change- genic- splice | 681728 |
| C>T | Change- Non coding RNA | 681762 |
| T>G | Splice- Non Synonymous | 681771 |
| CT>TC | Amino acid change- Non Synonymous | 681836-7 |
| C>A | Change-Non coding RNA | 682298 |
| T>G | Amino acid change- Synonymous | 682321 |
| G>A | Amino acid change- Synonymous | 683134 |
| T>C | Amino acid change- Synonymous | 683718 |
| T>T | Amino acid change- Synonymous | 683751 |
| A>G | Amino acid change- Non synonymous | 683796 |
