# Supplementary material for: Characterization of a virulence factor in Plasmodiophora brassicae, with molecular markers for identification
Source: PLoS One. 2023 Sep 14;18(9):e0289842. doi: 10.1371/journal.pone.0289842 (PMC10501564; doi:10.1371/journal.pone.0289842)
Supplement: S2 Table — (DOCX) [file pone.0289842.s009.docx]

Table S2. Software programs used to determine the location of gene 1971 of *Plasmodiophora brassicae*

| Tool name | Locations or features predicted | Score | Webserver |
| --- | --- | --- | --- |
| BaCelLo ​​[24] | Cytoplasm | 0.7 | http://cello.life.nctu.edu. tw/cello2go/ |
| BUSCA ​​[32] | Cytoplasm | 0.7 | http://busca.biocomp.unibo.it/ |
| DeepLoc-1.0 ​​[28] | Cytoplasm, Soluble | 0.34, 0.57 | https://services.healthtech.dtu.dk/ service.php?DeepLoc-1.0 |
| DeepMito ​​[26] | Not mitochondrial | 0.9 | http://busca.biocomp.unibo.it/ deepmito/ |
| DeepSig ​​[27] | Neither a signal sequence nor a membrane-spanning segment | 1.0 | https://deepsig.biocomp.unibo.it/ welcome/default/index |
| MULocDeep ​​[28] | Cytoplasm, cytoskeleton | NA | http://mu-loc.org/ |
| Phobius prediction ​​[29] | Cytoplasm | NA | https://www.ebi.ac.uk/Tools/pfa/phobius/ |
| SCLpred-MEM ​​[30] | Non-EMS (endomembrane system and secretory pathway) | 7 (0=lowest; 9=highest) | http://distilldeep.ucd.ie/SCLpred-MEM/ |
| TargetP-2.0 [31] | No signal peptide and no mitochondrial transfer peptide | 1.0 | https://services.healthtech.dtu.dk/services/TargetP-2.0/ |
| TMHMM ​​[33] | No transmembrane domains | NA | https://services.healthtech.dtu.dk/services/TMHMM-2.0/ |
